# Dietary Glyceryl Polyethylene Glycol Ricinoleate as an Additive to Improve Intestinal Health in Post-Weaning Piglets

**DOI:** 10.3390/ani15070983

**Published:** 2025-03-29

**Authors:** Julieta M. Decundo, Susana N. Dieguez, Guadalupe Martínez, Fabián A. Amanto, María L. Maté, Juan P. Lirón, Denisa S. Pérez Gaudio, Carolina P. Bianchi, Aurélie Montagnon, Alejandro L. Soraci

**Affiliations:** 1 Laboratorio de Toxicología, Departamento de Fisiopatología, Facultad de Ciencias Veterinarias, Universidad Nacional del Centro de la Provincia de Buenos Aires, Tandil 7000, Argentina; susadie@vet.unicen.edu.ar (S.N.D.); guadam@vet.unicen.edu.ar (G.M.); denisa@vet.unicen.edu.ar (D.S.P.G.); alejandro@vet.unicen.edu.ar (A.L.S.); 2 Facultad de Ciencias Veterinarias, Centro de Investigación Veterinaria de Tandil (CIVETAN, UNCPBA-CICPBA-CONICET), Tandil 7000, Argentina; cbianchi@vet.unicen.edu.ar; 3 Comisión de Investigaciones Científicas de la Provincia de Buenos Aires (CIC-PBA), La Plata 1900, Argentina; 4 Área de Producción Porcina, Departamento de Producción Animal, Facultad de Ciencias Veterinarias, Universidad Nacional del Centro de la Provincia, Tandil 7000, Argentina; famanto@vet.unicen.edu.ar; 5 Laboratorio de Farmacología, Centro de Investigación Veterinaria de Tandil (CIVETAN), UNCPBA-CONICET-CICPBA, Facultad de Ciencias Veterinarias, Universidad Nacional del Centro (FCV-UNCPBA), Tandil 7000, Argentina; mlmate@vet.unicen.edu.ar (M.L.M.); juanpedroliron@gmail.com (J.P.L.); 6 Laboratorio de Endocrinología, Centro de Investigación Veterinaria de Tandil (CIVETAN), UNCPBA-CONICET-CICPBA, Facultad de Ciencias Veterinarias, Universidad Nacional del Centro (FCV-UNCPBA), Tandil 7000, Argentina; 7 Orffa Additives B.V., 4817 ZL Breda, The Netherlands; montagnon@orffa.com

**Keywords:** pig, glyceryl polyethylene glycol ricinoleate, gastrointestinal tract, microbiota, weaning stress, emulsifier

## Abstract

Early weaning in intensive pig production induces stress, compromising gastrointestinal health. Poor fat digestion results from the piglets’ underdeveloped digestive system. Dietary emulsifiers can enhance fat utilization, and glyceryl polyethylene glycol ricinoleate (GPGR) has been shown to improve pig performance. This study evaluated GPGR’s effects on intestinal health in weaned piglets in a commercial production farm. A total of 380 just weaned (21 days old) piglets were divided in two groups of 190 animals each (in four replicates) that received either a basal diet (control) or a basal diet + 350 g/ton GPGR pharmaceutical formulation as top dress. Blood samples were collected at pre-established days, and intestinal sampling occurred 15 days post-weaning. Plasma cortisol, citrulline, intestinal morphology, mucus quality, enzymatic activity, volatile fatty acids (VFAs), and cecal microbiota were analyzed. GPGR did not alter plasma cortisol but increased citrullinemia (P: 0.024), suggesting greater enterocyte functional mass. GPGR piglets showed improved intestinal morphology (greater villus height, villus height:crypt depth ratio, and intestinal absorption area, *p* < 0.05) and higher enzymatic maltase activity (*p* ≤ 0.014). VFAs, bacterial adherence to mucus, and goblet cell counts were unaffected. Dietary GPGR increased *Firmicutes* and *Actinobacteria* (P: 0.014 and P: 0.045, respectively) while reducing *Proteobacteria* (*p* < 0.001). In conclusion, dietary GPGR promotes intestinal health in weaned piglets by improving epithelial structure, digestive function, and microbiota balance, representing a promising strategy to support piglets in overcoming the early nursery phase.

## Simple Summary

In intensive production systems piglets are weaned prematurely to improve productivity. Early weaning causes stress that directly damages intestinal health and ultimately results in suboptimal growth. Certain dietary additives have shown to improve fat digestion and nutrient absorption helping piglets overcome the negative consequences of early weaning. In our study we evaluated the effects of including an emulsifier, glyceryl polyethylene glycol ricinoleate (GPGR), in the diet of weaned piglets under commercial rearing. A total of 380 animals were assigned to either a control group (fed regular diet) or a GPGR group (fed regular diet supplemented with GPGR). We assessed the morphology of the intestinal epithelium, digestive enzymes activity, and gut microbiota composition as indicators of intestinal health. The piglets fed GPGR showed improved epithelial morphology, superior digestive enzymes activity, and a more balanced microbial community compared to control piglets. Our findings demonstrate that the incorporation of GPGR to the post-weaning diet supports intestinal health and can be used as nutritional strategy to enhance the adaptation of piglets to this challenging period.

## 1. Introduction

Modern pig production practices expose piglets to high stress during weaning due to multiple factors. Among the most important stressors, the abrupt shift from liquid, highly digestible, and extensively emulsified sow milk to a more complex and less digestible solid feed directly compromises gastrointestinal health. At the same time, immature digestive and immune systems and low feed intake after early weaning result in poor digestive enzymes activity, reduced mucins secretion, disrupted epithelial architecture, and imbalanced gut microbiota homeostasis [1,2,3]. A nutritional strategy to help piglets relieve the negative effects of early weaning and meet energy requirements is the inclusion of digestible fats, like vegetable oils, in the diet. However, during the acute phase of weaning stress, the lower levels of lipase production [3,4] and bile acids synthesis/secretion [5,6,7], as well as the reduced abundance of fatty acids transport proteins [8] hinder fat absorption. To enhance fat digestibility and promote its utilization as an energy source, recent research explores the use of exogenous emulsifiers. Studies using different emulsifiers—lysophospholipids, soy lecithin, lysolecithin, sodium stearoyl 2 lactylate, polyglycerol fatty acid esters, glyceryl polyethylene glycol ricinoleate, bile acids, etc.—demonstrate that their incorporation tends to increase digestibility and improve productive performance parameters such as average daily gain and feed conversion ratio even in low energy diets [9,10,11,12,13,14,15]. The extent of the observed effects depends on the age of the animals, fat source, type, and concentration of the emulsifier used, among other factors.

For the past few years, the use of glyceryl polyethylene glycol ricinoleate (GPGR) as an additive in pig diets has been gaining interest. GPGR is a non-ionic emulsifier that exhibits lower interfacial tension, higher hydrophilic–lipophilic balance, emulsion stability through the gastrointestinal tract, and high mixed micelle solubilization rendering a higher extent of lipid hydrolysis compared to other emulsifiers such as lysolecithin and monoolein [16,17]. Investigations on the effects of GPGR in pigs have prioritized growth performance and digestibility [10,14]. Expanding research to include this additive’s impact on gastrointestinal health would offer a comprehensive understanding of its potential benefits and drawbacks. Hence, the aim of the present study was to evaluate the impact of dietary GPGR on intestinal heath of weanling piglets. Our multifaceted approach examined stress, intestinal histomorphology, metabolism, and microbiota analysis.

## 2. Materials and Methods

This research was conducted on a 400-sow farrow-to-finish commercial farm with high health status, located in Buenos Aires Province, Argentina. All animals were subjected to the farm’s routine management practices.

### 2.1. Animals and Treatments

At weaning (22 ± 1.4 days of age), we randomly selected 380 piglets (46% females and 54% immuno-castrated males) of homogeneous weight (6.52 ± 1.26 kg), weaned from clinically healthy sows of the same commercial genetic line (Swine Genetic Branch; Choice Genetics Co, Rafaela, Argentina) and homogeneous reproductive performance (second or third parity, 15 ± 2 pigs born alive, 12.50 ± 2.09 mm back fat thickness by the end of gestation). The selected piglets were divided into two groups, and to minimize maternal influence each litter was equally represented in both groups. Control piglets (n = 190) received a basal diet that consisted of the following commercial feed: during the first week post weaning Perfecto Nursery) and during the second week post weaning Perfecto Transición, both provided by Biofarma S.A., Córdoba, Argentina). Nutritional specifications of each feed are presented in Table 1. The fat source in both commercial feeds were soy oil and bovine milk powder. Lysine contents and all other nutrient requirements were supplied in compliance with the National Research Council (NRC, 2012) [18]. GPGR piglets (n = 190) received a basal diet supplemented with 350 g/ton of an emulsifier formulation containing 21% glyceryl polyethylene glycol ricinolate and diatomaceous earth (Excential Energy Plus, Orffa Additives B.V., Breda, The Netherlands) incorporated as top dress. The trial was conducted in four replicates of 95 ± 2 animals each (47 ± 1 control piglets and 47 ± 1 GPGR piglets in each replicate). Control and GPGR piglets were housed in different pens within the same weaning room equipped with full slatted floors and a computerized ventilation system keeping a constant temperature of 29 °C for the first week after weaning and decreasing by 1 °C weekly. Each pen was furnished with a stainless-steel feeder and five nipple drinkers, providing ad libitum access to feed and water throughout the trial. Animal health status was monitored daily. Trained personnel recorded signs of diarrhea, behavioral changes, any observable abnormalities related to feed or water intake (such as refusal to eat or drink), growth (all piglets were weighed at weaning and at the end of the trial), and mortality following standard farm procedures.

### 2.2. Sample Collection and Processing

Forty piglets from each group (ten piglets from each replicate) were randomly selected and ear-tagged for blood and gastrointestinal sampling.

#### 2.2.1. Plasma

Blood samples were collected in heparinized tubes by venipuncture of anterior vena cava at weaning (day 0) and subsequently at 4, 8, 12, and 15 days later. Sampling began at 8:00 a.m. and concluded within a maximum of 30 min. Plasma was obtained after centrifugation and stored at −20 °C until analyzed.

Plasma cortisol concentrations were used as an indicator of stress [19,20]. An RIA kit (IM 1841, Beckman Coulter, Immunotech, Indianapolis, IN, USA) previously employed with pig plasma [21,22] was used to obtain plasma cortisol concentration. The assay’s sensitivity was 5 nmol/L and the coefficient of variation was 6.4% (between 20 and 2000 nmol/L). Samples were measured in duplicate.

Plasma citrulline concentrations were used as an indicator of intestinal integrity, reflecting enterocytes functional mass and metabolism [23,24,25]. Citrullinemia was analyzed after derivatization using o-phthalaldehyde followed by HPLC-FLD. A C18 chromatographic column maintained at 30 °C was used. The mobile phase consisted of (A) sodium acetate buffer 50 mmol/L (pH 6.8) and (B) methanol:acetonitrile (2:1), programmed in gradient elution mode. Excitation and emission wavelengths were 338 and 425 nm, respectively [26]. Method performance showed optimum linearity (r^2^ > 0.999) between 0.5 and 20 µmol/L, accuracy 2.09%, repeatability and intermediate precision < 10% for all concentrations.

#### 2.2.2. Gastrointestinal Tract

Fifteen days post weaning, twelve animals from each group (three animals from each replicate) were randomly selected from ear-tagged piglets and euthanized using captive bolt stunning followed by jugular bleeding.

The pH was measured in stomach, ileum, caecum, and colon using a pH meter (UP-25 Denver Instruments, Denver, CO, USA).

For histomorphological evaluation, 10-cm segments from the mid jejunum (1.5 m from stomach) and ileum (20 cm proximal to ileocecal valve) were collected, washed with saline, fixed in 10% neutral buffered formalin, and embedded in paraffin to be sliced and stained using hematoxylin & eosin (HE) and periodic acid-Schiff (PAS). HE-stained tissue slides were examined under light microscope (Olympus BX40, Olympus Corporation, Tokyo, Japan) equipped with an image processing program (ToupTekTM Toup ViewTM^®,^ Anji, Zhejiang, China) to obtain the length and width of 50 villi and their associated crypts from each histological section [21,27].

The mathematical model of Kisielinski (2002) [28] was used to estimate the intestinal absorption area (IAA). Villi height (Vh) to crypts depth (Cd) ratio (Vh:Cd) was used as a biomarker of intestinal atrophy [29]. Goblet cells in villi (vGC) and crypts (cGC), expressed as goblet cells/100 villi or crypts, were identified in PAS-stained tissue slides. All histological measurements were carried out by a single analyst who was unaware of the origin of the samples.

Mucus quality was evaluated by the ability of its glycoproteins to adhere to pathogenic *E. coli* following the method described by Bai et al. (2000) [30]. Briefly, ileum samples were opened along the mesenteric border and mucus was carefully scraped off to extract only the external fraction, collected into sterile tubes, and stored at −70 °C until analysis. The mucus was diluted in sterile saline solution and centrifuged. The supernatant was sterilized by filtration (0.22 µm filter membranes) to obtain glycoproteins responsible for bacteria adherence. The glycoproteins solution was incubated with *E. coli* O157:H7 (103 CFU/mL) at 37 °C under continuous agitation. Subsequent centrifugations rendered a pellet with adhered bacteria and a supernatant with non-adhered bacteria. Aliquots from both fractions were plated on MacConkey agar with Sorbitol (Britannia S.A., Ciudad Autónoma de Buenos Aires, Argentina) and incubated under aerobic conditions at 37 °C for 24 h. Colonies were then counted.

The enzymatic activity of intestinal brush border disaccharidases was measured to evaluate enterocyte maturity and nutrient digestive capacity [31,32]. Segments from duodenum, proximal jejunum (15 cm from stomach), mid jejunum (1.5 m from stomach), and ileum (20 cm proximal to ileocecal valve) were opened along the mesenteric border and washed with sterile saline solution to remove residual contents and mucus. The mucosa was carefully scrapped off using a scalpel. An amount of 1.00 g of mucosa was homogenized in sterile saline solution and cold centrifuged. The supernatant represented the crude enzyme solution. The Bradford method was used to determine protein concentration in homogenates. Sucrase, maltase, and lactase activities were quantified based on the amount of glucose released after incubation with sucrose, maltose, and lactose, respectively. The reaction products were then treated with the glucose oxidase-peroxidase reagent using O-dianisidine as a chromogen. Absorbance was measured using a Shimadzu RF5301PC molecular absorption spectrophotometer at 450 nm [33]. Enzymatic activity was expressed as U/mg of protein (where U represents the amount of enzyme that hydrolyses 1 mmol of lactose, sucrose, or maltose in 1 min under the standard assay conditions).

Volatile fatty acids (VFAs) were quantified following the method described by Jouany (1982) [34]. Briefly, 1.00 g of cecal content was collected in a sterile tube containing phosphoric acid and stored at −70 °C until analysis. VFAs were extracted with methanol and quantified by gas chromatography coupled to a Flame Ionization Detector (Shimadzu; model GC-17A, Kyoto, Japan). Chromatographic separation was achieved in a 19091N-133 INNOWAX 30 m capillary column (Agilent, Santa Clara, CA, USA). Calibration curves were performed using a mixture of volatile fatty acids standards (Supelco, Muskoka, ON, Canada) and 2-ethyl-butyric acid as internal standard (Fluka, Charlotte, NC, USA). Method performance showed optimum linearity (r^2^ > 0.995) within 0.0625–9 mmol/L. Accuracy, repeatability, and intermediate precision were less than 10% for all VFAs at all concentrations.

To perform microbiota analysis, the intestinal content from caecum was collected in sterile tubes and immediately stored at −72 °C until DNA extraction. Total bacterial DNA was extracted from the caecal content (0.20 g) using a Qiamp^®^PowerFecal^®^ Pro DNA kit (Qiagen, Redwood City, CA, USA). The quality and quantity of DNA were assessed using a Nanodrop spectrophotometer, and DNA samples were stored at −72 °C until analysis. The relative abundance and diversity of the bacterial community, as well as the composition in each caecum sample, were obtained through high-throughput sequencing. The V3–V4 hypervariable region of the bacterial 16S rRNA gene was PCR amplified using forward primer 341F (CCTAYGGGRBGCASCAG) and reverse primer 806R (GGACTACNNGGGTATCTAAT), and the obtained product was purified. Concentration and quality of each amplicon were measured by Qubit™ fluorometer (Thermo Fisher Sientific, Waltham, MA, USA) and Agilent Bioanalyzer 2100 (Santa Clara, CA, USA). Barcoded amplicons were sequenced using the Illumina NovaSeq 6000 platform (HiSeq, Novogene, Durham, NC, USA). The FASTQ files were imported into QIIME2, and the DADA2 plugin was applied to denoise and quality-filter the reads. A naïve Bayes classifier was trained against the SILVA v138 database—restricted to the V3–V4 region—to assign taxonomy to the sequences [35]. The OTU table, taxonomy, metadata, and phylogenetic tree were imported into the R package Phyloseq (version 1.42.0) [36]. Sequences identified as chloroplast, mitochondrial, or eukaryotic were removed. Library rarefaction was performed to calculate alpha and beta diversities among samples, and standardized to an even depth of 90% of the sample with the fewest reads.

### 2.3. Statistical Analysis

Results were expressed as mean ± standard deviation for each group. RStudio software version 4.2.2 was employed for the statistical analysis. Normality and homoscedasticity were tested using Shapiro–Wilks and Bartlett’s tests, respectively. Fisher’s test was used to analyze associations between groups and mortality percentage. Plasma cortisol and citrulline concentrations were analyzed by repeated measures ANOVA where treatment effect, sampling day effect, and their interaction were evaluated. When ANOVA resulted in statistically significant effects (*p* < 0.05), Tukey or Dunn tests, as applicable, were used to detect differences between treatments, sampling day, or their interactions. From plasma concentrations at different time points, the area under the concentration-time curves was calculated for citrulline (AUCcit) and cortisol (AUCcort) using PK Solution 2.0 software [37]. The variables ADG, AUCcit, AUCcort, IAA, Vh:Cd, vGC, cGC, the percentage adherence of bacteria to mucus, enzymatic activity of intestinal disaccharidases, and VFAs concentrations, were analyzed by Student’s T test or Mann–Whitney test, as applicable, to detect differences between groups. The intestinal zones were studied individually.

For microbiome analysis, alpha diversity was estimated using the Shannon index. Differences between groups in Shannon index were analyzed by the Kolmogorov–Smirnov test. Bray–Curtis and unweighted UniFrac distances were calculated to determine beta diversity, and ordination was performed through principal coordinate analysis (PCoA). The differential relative abundance of OTUs was determined using DESeq2 [38]. The Wald test was employed to determine group differences at the phylum, class, order, and family level (relative abundance values higher than 1% for phyla, class order, and bacterial families were considered for discussion). Padj < 0.05 indicates differences between groups.

## 3. Results

The animals remained healthy, with consistent feed and water consumption and no evidence of diarrhea throughout the trial. Both piglet groups showed similar daily weight gain (0.24 ± 0.09 kg/day for control and 0.25 ± 0.08 kg/day for GPGR; P: 0.140) and mortality (1.39% for control and 1.17% for the GPGR; P: 0.998) within the expected range for the first 15 days of the nursery stage.

### 3.1. Plasma

Plasma cortisol concentration was significantly influenced by sampling day (P: 0.001), but not by treatments (P: 0.556) or interactions between treatment and sampling day (P: 0.971). Plasma cortisol concentration peaked on day 4 and decreased towards pre-weaning values (day 0) by 8 days post weaning. Both piglet groups showed similar AUCcor (P: 0.794) (Table 2).

Plasma citrulline concentration was not affected by interactions between post-weaning day and treatment (P: 0.069), but it was significantly influenced by both sampling day (P: 0.001) and treatment (P: 0.024). Citrullinemia sharply decreased 4 and 8 days post weaning followed by a significant increase towards 12 and 15 post weaning without reaching pre-weaning (day 0) values. GPGR piglets showed higher citrullinemia than control piglets (Table 2). Moreover, GPGR piglets exhibited higher AUCcit compared to the control piglets (P: 0.042; Table 2).

### 3.2. Gastrointestinal Tract

Both control and GPGR piglets showed similar pH in each intestinal zone (*p* < 0.05). Mean pH ± SD were 3.12 ± 1.06, 7.07 ± 0.19, 5.96 ± 0.43, 6.10 ± 0.55 for stomach, ileum, caecum, and colon, respectively.

Regarding the histomorphological analysis, GPGR piglets exhibited higher Vh, Vh:Cd, and IAA in jejunum and ileum than control piglets. Both groups showed similar bacterial adherence to mucus (mucus quality) and similar Cd, vGC, and cGC in jejunum and ileum (Table 3).

Lactase activity was similar for control and GPGR piglets in all intestinal zones (Table 4). Sucrase activity was similar for control and GPGR piglets in proximal jejunum, mid jejunum, and ileum. In duodenum, the GPGR piglets showed higher sucrase activity than the control piglets (Table 3). Maltase activity was consistently higher in the GPGR piglets compared to the control piglets in all intestinal zones (Table 4).

Cecal VFA concentrations (acetic, propionic, butyric, valeric, and total) were similar in both control and GPGR piglets (Table 5).

**Figure 1:**
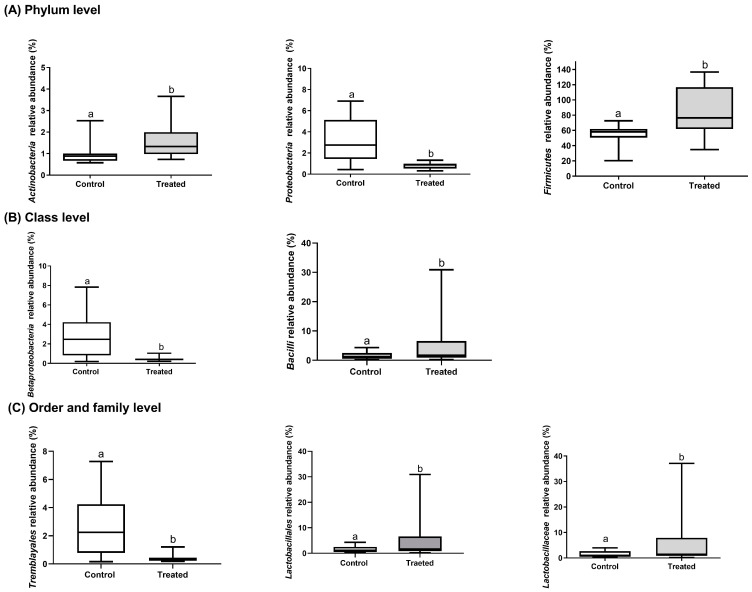
Relative abundance (%) of control and GPGR piglets for (**A**) *Actinobacteria*, *Proteobacteria*, and *Firmicutes* (phylum level); (**B**) *Betaproteobacteria* and *Bacilli* (class level); (**C**) *Tremblayales* and *Lactobacillales* (order level); and *Lactobacillaceae* family. Different letters indicate statistically significant differences (*p* < 0.05).

The quality report of microbiome analysis demonstrated that the library size ranged between 149,075 and 178,704 clean reads per sample. All the samples were adequate for bioinformatics analyses (Table S1). The most abundant phyla in the cecal microbiome of both control and GPGR piglets were *Firmicutes* (69.93%), *Bacteroidetes* (26.30%), *Proteobacteria* (1.99%), and *Actinobacteria* (1.25%). At family level, the most abundant were *Lachnospiraceae* (26.61%), *Prevotellaceae* (20.36%), *Ruminococcaceae* (16.02%), *Erysipelotrichaceae* (7.90%), *Clostridiaceae* (6.89%), *Lactobacillaceae* (4.87%), *Veillonellaceae* (4.77%), *Pararevotellaceae* (3.79%), and *Coriobacteriaceae* (1.15%). Estimates of alpha diversity showed a similar Shannon index for both piglet groups (control 4.78 ± 0.29 and GPGR 4.90 ± 0.23; P: 0.536) (Figure S1). Related to beta diversity, the PCoA plots revealed overlapping group cluster patterns (Figure S2), indicating similar microbiome structure in both piglet groups. Relative abundance analysis demonstrated that GPGR piglets exhibited higher relative abundance of *Firmicutes* (Log2FC: 0.6, Padj: 0.014) and *Actinobacteria* phyla (Log2FC: 0.7, Padj: 0.045), *Bacilli* class (Log2FC: 2.6, Padj: 0.001), *Lactobacillales* order (Log2FC: 2.6, Padj: 0.002), and *Lactobacillaceae* family (Log2FC: 2.4, Padj: 0.009), while exhibiting lower *Proteobacteria* phylum (Log2FC = −2, Padj = < 0.001), *Betaproteobacteria* class (Log2FC = −2.6, Padj < 0.001), and *Tremblayales* order (Log2FC = −2.7, Padj < 0.001) than control piglets (Figure 1). The relative abundances of the remaining bacteria at the phylum, class, order, and family levels were similar between piglet groups (*p* > 0.05).

## 4. Discussion

Since the intensification of pig production worldwide, which introduced early weaning as a common practice, investigations have focused on the optimization of post-weaning diets, with special attention on the incorporation of fats in order to meet the energy requirements of young piglets. But the underdeveloped digestive system of piglets at this stage may render poor fat digestion causing a negative impact on intestinal health and zootechnical parameters. To improve fat digestion, the incorporation of emulsifiers to post-weaning diets have shown promising results, especially regarding growth performance [10,11,14,15]. However, research on the impact of emulsifiers on intestinal health remains limited. Therefore, in the present work we studied the effects of a specific dietary GPGR (which is extensively used as emulsifier in intensive productions) on the intestinal health of post weaned piglets following a holistic approach that integrates physiological, morphological, and microbiological aspects of the intestinal tract. Moreover, our study was conducted on a commercial farm to provide practical implications for pig production.

Newly weaned piglets experience significant stress due to the abrupt transition to a novel environment, routine, diet, and social group. While individual responses may vary widely, increased plasma cortisol levels are a consistent indicator of stress and can be used as a reliable biomarker [20,25]. In the present study, both piglet groups exhibited similar plasma cortisol concentration along the sampling period, indicating that the inclusion of dietary GPGR did not elicit additional stress. This finding is reinforced by the comparable AUCcor values observed between control and GPGR piglets. On the other hand, plasma cortisol levels peaked 4 days post weaning reflecting that the animals were undergoing the acute phase of weaning stress which can persist for about one week [1,25]. Thereafter, cortisol concentration decreased to reach pre-weaning (day 0) values and remained within the expected range for post weaned piglets [19].

Plasma citrulline concentrations are positively correlated with enterocytes mass and intestinal metabolism [23,24]. In the current work, both piglet groups displayed similar citrullinemia profiles: citrullinemia peaked pre-weaning (day 0) and sharply decreased over the first 4 to 8 days post weaning, subsequently increasing but not returning to pre-weaning values even after 15 days. As it has been shown in several studies, this pattern is indicative of the detrimental impact of weaning stress on intestinal function and the subsequent recovery process as the piglets adapt to their new situation [3,25,39]. At the same time, the GPGR piglets consistently exhibited higher citrullinemia levels than control piglets throughout the sampling period. Thus, the treatment elicited a significant effect, resulting in increased mean plasma citrulline concentrations and overall citrulline production (AUCcit) during the first 15 days of the nursery stage. This effect may be explained by the protective role of emulsifiers against oxidative stress—mainly lipid peroxidation—favoring intestinal development and metabolism [14,16,40,41]. To our knowledge, this is the first study to establish the effect of a dietary emulsifier on citrullinemia in pigs.

Gastrointestinal pH values were similar in both piglet groups and fell within the expected range considering the intestinal portions and the age of the study animals [21,42], which shows that GPGR did not modify the pH of the gastrointestinal tract.

Histomorphological analysis from our study revealed that dietary GPGR improved intestinal structure, indicated by greater Vh, higher Vh:Cd, and increased IAA in GPGR piglets compared to controls. This effect has been previously described in pigs fed diets supplemented with different emulsifiers [12,43,44] and can be attributed to enhanced nutrients absorption, especially fats and lipophilic vitamins [43].

In addition, oxidative stress and lipid peroxidation—involved in the inflammatory processes, characteristic of the post-weaning period—can disrupt cellular redox balance, impair intestinal turnover, and cause intestinal atrophy [45,46,47]. In the present work, the reduced intestinal atrophy, represented by higher Vh:Cd, IAA, and citrulline production exhibited by the GPGR piglets compared to the control piglets indicate that this emulsifier may prevent oxidative stress and lipid peroxidation [40,41].

Intestinal mucus quality, which was evaluated by its ability to bind pathogenic *E. coli*, is generally correlated with the number of Goblet cells present in villi and crypts. Even if other studies have shown that exposure to emulsifiers impair mucus structure [48], our results revealed no impact of dietary GPGR on either Goblet cells counts or mucus quality. Similarly, Kubis et al. (2020) [49] reported that ileal Goblet cell counts remained constant following the inclusion of GPGR in the diet of broiler chickens.

After weaning, brush border enzymes, represented by disaccharidases in our study, are toughly modified in response to dietary changes. In this way, lactase activity decreases due to the reduction in milk and dairy derivatives consumption along the nursery stage while sucrase and maltase increase due to the introduction of cereals in the diet [50]. In the present work, lactase activity was unaffected by dietary incorporation of GPGR, probably due to the relatively high concentrations of dairy derivatives in the diet during the early post-weaning period. Meanwhile, dietary GPGR significantly increased the activity of maltase in all intestinal segments and that of sucrase in the duodenum. These results suggest that GPGR piglets have better nutrient digestibility and absorption, leading to improved feed adaptation after weaning [13].

Improved nutrient digestibility and fat absorption due to emulsifiers like GPGR reduce the substrate available for fat-fermenting bacteria in the large intestine, leading to a lower concentration of VFAs in the cecum [51]. However, in our study we found similar concentrations of individual and total VFA in both control and GPGR piglets. Our results agree with those of Camp Montoro et al. (2022) [52], who found no differences in VFA production between pigs receiving low and high energy diets (with and without addition of a fat source, respectively).

The microbiome analysis in our work showed that the predominant phyla observed aligned with findings from previous studies [43,53,54,55]. No significant differences in alpha diversity were observed between groups, with values around 4.8, which are consistent with other studies for the cecal microbiome of weanling piglets [54,56]. Analysis of beta diversity evidenced similar microbiome structure in both piglet groups. Nonetheless, GPGR supplementation to piglets’ diets demonstrated effects on the relative abundance of certain taxonomic levels within the cecal microbiome.

Differences in relative abundance of bacteria may be responsible for intestinal and general health traits. *Firmicutes* and *Actinobacteria*, which in the present study were more abundant in the GPGR piglets than in the controls, are typically associated with efficient energy extraction from fats and complex carbohydrates in the hind gut [57]. At the same time, the production of metabolites through fermentation renders intestinal benefits to the host, for example, providing energy to colonocytes, maintaining pH balance, and modulating inflammatory responses. Different studies have shown better feed efficiency, reduced inflammatory conditions, and overall health improvement in piglets that exhibit high relative abundance of these phyla in cecum or feces [58,59].

Additionally, GPGR piglets showed a higher relative abundance of *Lactobacillaceae* compared to the control piglets. Members of this family are known for beneficial functions, including pathogen protection, intestinal microbiome balance and digestion, oxidative stress regulation [13,54], and adjustment of the host epigenome [60]. On the other hand, the lower abundance of *Proteobacteria* (especially pathogenic species of the phylum like *Escherichia* or *Salmonella*) may have exerted a beneficial effect as they are often associated with gut dysbiosis and inflammation as they proliferate in an imbalanced gut environment [61].

The higher relative abundance of *Firmicutes* along with lower relative abundance of *Proteobacteria* (class *Betaproteobacteria*, order *Tremblayales*) in GPGR compared to control piglets in our study suggest that the consumption of GPGR during the early nursery period results in a healthier and more balanced gut microbiota, reducing the risk of inflammation, pathogenic infections, and gut disorders [62,63]. These inferences are supported by our previously described results on intestinal health parameters, including citrullinemia, villus height over crypts depth ratio, intestinal absorption area, and brush border enzymes activity, all of which were more favorable in GPGR-treated piglets than in the control group. Conversely, cecal concentrations of volatile fatty acids did not differ between groups, likely because the treatments did not cause significant changes in bacteria species that produce these acids.

## 5. Conclusions

Our study demonstrates that the incorporation of GPGR into the post-weaning diet provides significant intestinal health benefits, including enhanced enterocyte metabolism during the acute phase of weaning stress, improved mucosal architecture, and modulation of cecal microbiota. Thus, specific dietary GPGR represents a promising strategy to support piglets in overcoming the challenges of the early nursery stage.

## Figures and Tables

**Table 1:** Nutritional specifications of basal diets fed to control and GPGR piglets.

Nutritional Specifications	Feed First Week	Feed Second Week
Dry Matter (%)	92.50	92.50
Crude protein (%)	21.85	20.56
Fat (%)	5.80	6.53
Starch (%)	25.80	31.29
Crude Fiber (%)	1.50	1.65
Ash (%)	5.49	5.35
Calcium	0.74	0.90
Available phosphorus (%)	0.57	0.48
Metabolizable Energy (kcal)	3394.41 Kcal	3410.40 Kcal
Net Energy (kcal)	2436.35 Kcal	2489.17 Kcal
Lactose (%)	12.60	10.54
Digestible lysine (%)	1.52	1.39
Digestible methionine (%)	0.61	0.46
Methionine + cystine (%)	0.94	0.59
Digestible threonine (%)	0.98	0.84
Digestible tryptophan (%)	0.29	0.24
Digestible arginine (%)	1.28	1.27
Digestible valine (%)	0.98	0.92
Digestible isoleucin (%)	0.82	-
Digestible leucine (%)	1.61	-

**Table 2:** Mean plasma citrulline and cortisol concentrations for different sampling days (0, 4, 8, 12, 15) and piglet groups (Control and GPGR).

Plasma		Piglets		Day Mean
Concentrations	Days	Control	GPGR	
Cortisol (nmol/L)				
	0	184.30 ± 92.38	205.08 ± 132.13	201.90 ± 112.63 ^a^
	4	208.46 ± 68.04	225.64 ± 70.85	217.05 ± 69.62 ^b^
	8	194.59 ± 70.31	179.66 ± 62.74	187.13 ± 67.92 ^ab^
	12	174.88 ± 51.83	168.96 ± 54.92	171.91 ± 53.14 ^a^
	Treatment mean	192.26 ± 72.09	194.93 ± 89.52	
	AUCcor	2344.26 ± 526.86	2384.59 ± 601.88	
Citrulline (µmol/L)				
	0	57.69 ± 23.56	69.30 ± 28.75	63.22 ± 26.65 ^a^
	4	33.75 ± 11.44	35.56 ± 12.00	34.62 ± 11.67 ^b^
	8	30.34 ± 10.98	33.54 ± 13.88	31.88 ± 12.49 ^b^
	12	40.31 ± 19.53	50.85 ± 20.22	45.40 ± 20.44 ^c^
	15	37.15 ± 17.15	46.57 ± 17.81	41.39 ± 17.93 ^c^
	Treatment mean	39.85 ± 19.55 ^a^	47.23 ± 23.44 ^b^	
	AUCcit	563.82 ± 205.97 ^a^	661.85 ± 209.97 ^b^	

Different superscript letters within rows and superscript letters within the column “Mean” indicate statistically significant differences (*p* < 0.05).

**Table 3:** Histomorphological variables: villi height (Vh), crypts depth (Cd), villi height to crypts depth ratio (Vh:Cd), intestinal absorptive area (IAA), number of goblet cells/100 villi (vGC) and number of goblet cells/100 crypts (cGC), adherence of bacteria to mucus as percentage (adherence %) for control and GPGR piglets analyzed in jejunum and ileum. Results are expressed as mean ± SD. Different superscript letters within rows indicate statistically significant differences (*p* < 0.05).

		Piglets		
Zone	Variables	Control	GPGR	*p*-Value
Jejunum				
	Vh (μm)	299.54 ± 34.62 ^a^	379.02 ± 57.95 ^b^	<0.001
	Cd (μm)	99.52 ± 8.64	105.21 ± 15.73	0.241
	Vh:Cd	3.02 ± 0.37 ^a^	3.64 ± 0.62 ^b^	0.007
	IAA	5.83 ± 0.66 ^a^	6.71 ± 0.62 ^b^	0.006
	vGC	834.13 ± 144.59	1002.46 ± 343.84	0.219
	cGC	1156.58 ± 373.66	968.94 ± 154.09	0.122
Ileum				
	Vh (μm)	251.72 ± 42.32 ^a^	300.39 ± 33.21 ^b^	0.005
	Cd (μm)	99.10 ± 9.02	96.54 ± 6.65	0.437
	Vh:Cd	2.55 ± 0.40 ^a^	3.12 ± 0.41 ^b^	<0.001
	IAA	4.86 ± 0.66 ^a^	5.47 ± 0.74 ^b^	0.044
	vGC	1600.83 ± 220.18	1482.67 ± 344.49	0.088
	cGC	1022.08 ± 298.00	1354.96 ± 509.14	0.328
	Adherence (%)	90.08 ± 6.20	93.45 ± 3.70	0.325

**Table 4:** Mean enzymatic activity of sucrase, lactase, and maltase for control and GPGR piglets in different GIT zones (duodenum, proximal jejunum, mid jejunum, and ileum). Different superscript letters within rows indicate statistically significant differences (*p* < 0.05).

		Piglets		
Disaccharidases	Zone	Control	GPGR	*p*-Value
Sucrase (U/mg)				
	Duodenum	80.01 ± 44.97 ^a^	158.24 ± 109.82 ^b^	0.013
	Proximal jejunum	203.01 ± 137.27	248.09 ± 146.75	0.478
	Mid jejunum	1378.66 ± 498.76	1220.89 ± 425.25	0.413
	Ileum	485.37 ± 339.47	565.00 ± 417.56	0.671
Lactase (U/mg)				
	Duodenum	489.72 ± 203.06	419.46 ± 314.40	0.478
	Proximal jejunum	782.97 ± 283.25	667.66 ± 345.99	0.178
	Mid jejunum	2238.15 ± 819.07	1825.63 ± 870.00	0.244
	Ileum	119.21 ± 63.65	145.46 ± 119.90	0.551
Maltase (U/mg)				
	Duodenum	572.27 ± 393.39 ^a^	1351.15 ± 708.17 ^b^	<0.001
	Proximal jejunum	648,01 ± 283.62 ^a^	1860.62 ± 958.63 ^b^	<0.001
	Mid jejunum	1579.36 ± 631.36 ^a^	3817.85 ± 1328.14 ^b^	<0.001
	Ileum	1370.43 ± 886.72 ^a^	2405.74 ± 1007.43 ^b^	0.014

**Table 5:** Mean volatile fatty acids (VFAs) concentrations for control and GPGR piglets.

	Piglets		
VFA (mmol/L)	Control	GPGR	*p*-Value
Acetic	60.60 ± 19.38	64.18 ± 15.45	0.625
Propionic	21.29 ± 6.32	26.08 ± 6.72	0.086
Butyric	7.63 ± 3.55	7.50 ± 2.83	0.921
Valeric	1.09 ± 0.59	1.26 ± 1.32	0.792
Total	90.62 ± 27.87	99.02 ± 22.02	0.421

## Data Availability

The original contributions presented in this study are included in the article/Supplementary Material. Further inquiries can be directed to the corresponding author.

## Supplementary Materials

The following supporting information can be downloaded at: https://www.mdpi.com/article/10.3390/ani15070983/s1, Table S1: Quality report of microbiome analysis; Figure S1: Shannon diversity for control and GPGR piglets, Figure S2: Principal coordinates analysis (PCoA) plots based on (A) Bray-Curtis and (B) unweighted UniFrac distance metrics for control and GPGR piglets.

## References

[1] Campbell J.M., Crenshaw J.D., Polo J. (2013). The biological stress of early weaned piglets. J. Anim. Sci. Biotechnol..

[2] Heo J.M., Opapeju F.O., Pluske J.R., Kim J.C., Hampson D.J., Nyachoti C.M. (2013). Gastrointestinal health and function in weaned pigs, a review of feeding strategies to control post-weaning diarrhoea without using in-feed antimicrobial compounds. J. Anim. Physiol. Anim. Nutr..

[3] Tang X., Xiong K., Fang R., Li M. (2022). Weaning stress and intestinal health of piglets: A review. Front. Immunol..

[4] Hedemann M.S., Jensen B.B. (2004). Variations in enzyme activity in stomach and pancreatic tissue and digesta in piglets around weaning. Arch. Anim. Nutr..

[5] Jones D.B., Hancock J.D., Harmon D.L., Walker C.E. (1992). Effects of exogenous emulsifiers and fat sources on nutrient digestibility, serum lipids, and growth performance in weanling pigs. J. Anim. Sci..

[6] Lewis D.S., Oren S., Wang X., Moyer M.L., Beitz D.C., Knight T.J., Mott G.E. (2000). Developmental changes in cholesterol 7α-and 27-hydroxylases in the piglet. J. Anim. Sci..

[7] Price K.L., Lin X., Van Heugten E., Odle R., Willis G., Odle J. (2013). Diet physical form, fatty acid chain length, and emulsification alter fat utilization and growth of newly weaned pigs. J. Anim. Sci..

[8] He Y., Liu N., Ji Y., Tso P., Wu Z. (2022). Weaning stress in piglets alters the expression of intestinal proteins involved in fat absorption. J. Nutr..

[9] Bai G., He W., Yang Z., Fu H., Qiu S., Gao F., Shi B. (2019). Effects of different emulsifiers on growth performance, nutrient digestibility, and digestive enzyme activity in weanling pigs. J. Anim. Sci..

[10] Bontempo V., Comi M., Jiang X.R. (2016). The effects of a novel synthetic emulsifier product on growth performance of chickens for fattening and weaned piglets. Animal.

[11] Van Kinh L., Vasanthakumari B.L., Sugumar C., Thanh H.L.T., Van Thanh N., Wealleans A.L., Ngoan L.D., Loan N.V.T.H. (2022). Effect of a Combination of Lysolecithin, Synthetic Emulsifier and Monoglycerides on the Apparent Ileal Digestibility, Metabolizable Energy and Growth Performance of Growing Pigs. Animals.

[12] Song M., Zhang F., Chen L., Yang Q., Su H., Yang X., He H. (2021). Dietary chenodeoxycholic acid improves growth performance and intestinal health by altering serum metabolic profiles and gut bacteria in weaned piglets. Anim. Nutr..

[13] Sun H.Y., Kim I.H. (2019). Evaluation of an emulsifier blend on growth performance, nutrient digestibility, blood lipid profiles, and fecal microbial in growing pigs fed low energy density diet. Livest. Sci..

[14] Udomprasert P., Rukkwamsuk T. (2006). Effect of an exogenous emulsifier on growth performance in weanling pigs. Agric. Nat. Resour..

[15] Zhao P.Y., Li H.L., Hossain M.M., Kim I.H. (2015). Effect of emulsifier (lysophospholipids) on growth performance, nutrient digestibility and blood profile in weanling pigs. Anim. Feed. Sci. Technol..

[16] Ganna S., Abdel-Latif M., Ahmed H. (2022). Effect of Dietary Supplementation of Some Emulsifiers on Growth Performance, Carcass Traits, Lipid Peroxidation and Some Nutrients Digestibility in Broiler Chickens. Damanhour J. Vet. Sci..

[17] Michels D., Verkempinck S.H., Staes E., Spaepen R., Vermeulen K., Wealleans A., Grauwet T. (2023). Unravelling the impact of emulsifier blends on interfacial properties and in vitro small intestinal lipolysis of oil-in-water emulsions. Food Hydrocoll..

[18] National Research Council, Division on Earth and Life Studies, Board on Agriculture and Natural Resources, Committee on Nutrient Requirements of Swine (2012). Nutrient Requirements of Swine.

[19] Li L.A., Yang J.J., Li Y., Lv L., Xie J.J., Du G.M., Jiao X.L. (2016). Effect of weaning age on cortisol release in piglets. Genet. Mol. Res..

[20] Martínez-Miró S., Tecles F., Ramón M., Escriban D., Hernánde F., Madrid J., Cerón J.J. (2016). Causes, consequences and biomarkers of stress in swine, an update. BMC Vet. Res..

[21] Dieguez S.N., Decundo J.M., Martínez G., Amanto F.A., Bianchi C.P., Pérez Gaudio D.S., Soraci A.L. (2022). Effect of dietary oregano (*Lippia origanoides*) and clover (*Eugenia caryophillata*) essential oils’ formulations on intestinal health and performance of pigs. Planta Medica.

[22] Pluschke A.M., Williams B.A., Zhang D., Anderson S.T., Roura E., Gidley M.J. (2018). Male grower pigs fed cereal soluble dietary fibers display biphasic glucose response and delayed glycaemic response after an oral glucose tolerance test. PLoS ONE.

[23] Berkeveld M., Langendijk P., Verheijden J.H.M., Taverne M.A.M., Van Nes A., Van Haard P., Koets A.P. (2008). Citrulline and intestinal fatty acid-binding protein, Longitudinal markers of postweaning small intestinal function in pigs?. J. Anim. Sci..

[24] Crenn P., Messing B., Cynober L. (2008). Citrulline as a biomarker of intestinal failure due to enterocyte mass reduction. Clin. Nutr..

[25] Soraci A.L., Decundo J.M., Dieguez S.N., Martínez G., Pérez Gaudio D.S., Amanto F.A. (2023). Citrullinemia is a suitable biomarker for post weaning performance in piglets under intensive farming. J. Am. Vet. Med. Assoc..

[26] Wu G., Meininger C.J. (2008). Analysis of citrulline, arginine, and methylarginines using high-performance liquid chromatography. Methods Enzymol..

[27] Ohara T.E., Colonna M., Stappenbeck T.S. (2022). Adaptive differentiation promotes intestinal villus recovery. Dev. Cell.

[28] Kisielinski K., Willis S., Prescher A., Klosterhalfen B., Schumpelick V. (2002). A simple new method to calculate small intestine absorptive surface in the rat. Clin. Exp. Med..

[29] Canal A.M., Cubillos V., Zamora J., Reinhardt G., Paredes E., Ildefonso R., Alberdi A. (1999). Lesiones macro y microscópicas de intestino delgado de cerdos neonatos sin calostrar inoculados experimentalmente con cepas de *E. coli* fimbriadas. Arch. Med. Vet..

[30] Bai X., Liu X., Su Y. (2000). Inhibitory effects of intestinal mucus on bacterial adherence to cultured intestinal epithelial cells after surface burns. Chin. Med. J..

[31] Pluske J.R., Williams I.H., Aherne F.X. (1996). Maintenance of villous height and crypt depth in piglets by providing continuous nutrition after weaning. Anim. Sci..

[32] Solaymani-Mohammadi S., Singer S.M. (2011). Host immunity and pathogen strain contribute to intestinal disaccharidase impairment following gut infection. J. Immunol..

[33] Dahlqvist A. (1964). Method for Assay of Intestinal Disaccharidases. Anal. Biochem..

[34] Jouany J.P. (1982). Volatlile fatty acid and alcohol determination in digestive contents, silage juices, bacterial cultures and anaerobic fermentor contents. Sci. Aliment..

[35] Quast C., Pruesse E., Yilmaz P., Gerken J., Schweer T., Yarza P., Peplies J., Glöckner F.O. (2012). The SILVA ribosomal RNA gene database project, improved data processing and web-based tools. Nucleic Acids Res..

[36] McMurdie P.J., Holmes S. (2013). phyloseq, an R package for reproducible interactive analysis and graphics of microbiome census data. PLoS ONE.

[37] Farrier D. (1997). PK Solutions. Non Compartimental Pharmacokinetics Data Analysis.

[38] Love M.I., Huber W., Anders S. (2014). Moderated Estimation of Fold Change and Dispersion for RNA-Seq Data with DESeq2. Genome Biol..

[39] Lallès J.P., Bosi P., Smidt H., Stokes C.R. (2007). Weaning—A challenge to gut physiologists. Livest. Sci..

[40] Saleh A.A., Amber K.A., Mousa M.M., Nada A.L., Awad W., Dawood M.A., Abd El-Moneim A.E.M.E. (2020). A mixture of exogenous emulsifiers increased the acceptance of broilers to low energy diets, Growth performance, blood chemistry, and fatty acids traits. Animals.

[41] Siyal F.A., El-Hack M.E., Alagawany M., Wang C., Wan X., He J., Wang M., Zhang L., Zhong X., Wang T. (2017). Effect of soy lecithin on growth performance, nutrient digestibility and hepatic antioxidant parameters of broiler chickens. CABI Compendium.

[42] Franklin M.A., Mathew A.G., Vickers J.R., Clift R.A. (2002). Characterization of microbial populations and volatile fatty acid concentrations in the jejunum, ileum, and cecum of pigs weaned at 17 vs. 24 days of age. J. Anim. Sci..

[43] Li L., Wang H., Zhang N., Zhang T., Ma Y. (2022). Effects of α-glycerol monolaurate on intestinal morphology, nutrient digestibility, serum profiles, and gut microbiota in weaned piglets. J. Anim. Sci..

[44] Mitchaothai J., Yuangklang C., Vasupen K., Wongsuthavas S., Beynen A.C. (2010). Effect of dietary calcium and lecithin on growth performance and small intestinal morphology of young wild pigs. Livest. Sci..

[45] Li B., Zhang X., Zhang Q., Zheng T., Li Q., Yang S., Shao J., Guan W., Zhang S. (2024). Nutritional strategies to reduce intestinal cell apoptosis by alleviating oxidative stress. Nutr. Rev..

[46] Rosero D.S., Odle J., Moeser A.J., Boyd R.D., van Heugten E. (2015). Peroxidised dietary lipids impair intestinal function and morphology of the small intestine villi of nursery pigs in a dose-dependent manner. Br. J. Nutr..

[47] Zhu L., Cai X., Guo Q., Chen X., Zhu S., Xu J. (2013). Effect of N-acetyl cysteine on enterocyte apoptosis and intracellular signalling pathways’ response to oxidative stress in weaned piglets. Br. J. Nutr..

[48] Lock J.Y., Carlson T.L., Wang C.M., Chen A., Carrier R.L. (2018). Acute exposure to commonly ingested emulsifiers alters intestinal mucus structure and transport properties. Sci. Rep..

[49] Kubiś M., Kołodziejski P., Pruszyńska-Oszmałek E., Sassek M., Konieczka P., Górka P., Flaga J., Katarzyńska-Banasik D., Hejdysz M., Wiśniewska Z. (2020). Emulsifier and xylanase can modulate the gut microbiota activity of broiler chickens. Animals.

[50] Kelly D., Smyth J.A., McCracken K.J. (1991). Digestive development of the early-weaned pig, 1. Effect of continuous nutrient supply on the development of the digestive tract and on changes in digestive enzyme activity during the first week post-weaning. Br. J. Nutr..

[51] Blachier F. (2023). Metabolism of dietary substrates by intestinal bacteria and consequences for the host intestine. Metabolism of Alimentary Compounds by the Intestinal Microbiota and Health.

[52] Camp Montoro J., Solà-Oriol D., Muns R., Gasa J., Llanes N., Manzanilla E.G. (2022). Blood and faecal biomarkers to assess dietary energy, protein and amino acid efficiency of utilization by growing and finishing pigs. Porc. Health Manag..

[53] Ángel-Isaza J.A., Herrera Franco V., López-Herrera A., Parra-Suescun J.E. (2024). Nutraceutical Additives Modulate Microbiota and Gut Health in Post-Weaned Piglets. Vet. Sci..

[54] Guevarra R.B., Hong S.H., Cho J.H., Kim B.R., Shin J., Lee J.H., Kang B.N., Kim Y.H., Wattanaphansak S., Isaacson R.E. (2018). The dynamics of the piglet gut microbiome during the weaning transition in association with health and nutrition. J. Anim. Sci. Biotechnol..

[55] Wang L., Zou L., Li J., Yang H., Yin Y. (2021). Effect of dietary folate level on organ weight, digesta pH, short-chain fatty acid concentration, and intestinal microbiota of weaned piglets. J. Anim. Sci..

[56] Gresse R., Chaucheyras Durand F., Dunière L., Blanquet-Diot S., Forano E. (2019). Microbiota composition and functional profiling throughout the gastrointestinal tract of commercial weaning piglets. Microorganisms.

[57] Flint H.J., Scott K.P., Duncan S.H., Louis P., Forano E. (2012). Microbial degradation of complex carbohydrates in the gut. Gut Microbes.

[58] McCormack U.M., Curião T., Buzoianu S.G., Prieto M.L., Ryan T., Varley P., Crispie F. (2017). Exploring a possible link between the intestinal microbiota and feed efficiency in pigs. Appl. Environ. Microbiol..

[59] Mulder I.E., Schmidt B., Stokes C.R., Lewis M., Bailey M., Aminov R.I., Prosser J.I. (2009). Environmentally-acquired bacteria influence microbial diversity and natural innate immune responses at gut surfaces. BMC Biol..

[60] Qin Y., Roberts J.D., Grimm S.A., Lih F.B., Deterding L.J., Li R., Chrysovergis K. (2018). An obesity-associated gut microbiome reprograms the intestinal epigenome and leads to altered colonic gene expression. Genome Biol..

[61] Shin N.R., Whon T.W., Bae J.W. (2015). Proteobacteria, microbial signature of dysbiosis in gut microbiota. Trends Biotechnol..

[62] Lavelle A., Lennon G., O’Sullivan O., Docherty N., Balfe A., Maguire A., Mulcahy H.E., Doherty G., O’Donoghue D., Hyland J. (2015). Spatial variation of the colonic microbiota in patients with ulcerative colitis and control volunteers. Gut.

[63] Morgan X.C., Tickle T.L., Sokol H., Gevers D., Devaney K.L., Ward D.V., Reyes J.A., Shah S.A., LeLeiko N., Snapper S.B. (2012). Dysfunction of the intestinal microbiome in inflammatory bowel disease and treatment. Genome Biol..

